# Nontuberculous Mycobacterial Infections in a French Hospital: A 12-Year Retrospective Study

**DOI:** 10.1371/journal.pone.0168290

**Published:** 2016-12-13

**Authors:** Peggy Blanc, Hervé Dutronc, Olivia Peuchant, Frédéric-Antoine Dauchy, Charles Cazanave, Didier Neau, Gaëtane Wirth, Jean-Luc Pellegrin, Philippe Morlat, Patrick Mercié, José-Manuel Tunon-de-Lara, Marie-Sylvie Doutre, Philippe Pélissier, Michel Dupon

**Affiliations:** 1 Service de maladies infectieuses et tropicales, Centre Hospitalier Universitaire de Bordeaux, Bordeaux, France; 2 Laboratoire de Bactériologie, Centre Hospitalier Universitaire de Bordeaux, Bordeaux, France; 3 Université de Bordeaux, Infections humaines à mycoplasmes et à chlamydiae, Bordeaux, France; 4 INRA, Infections humaines à mycoplasmes et à chlamydiae, Bordeaux, France; 5 Service de médecine interne, Centre Hospitalier Universitaire de Bordeaux, Bordeaux, France; 6 Service de pneumologie, Centre Hospitalier Universitaire de Bordeaux, Bordeaux, France; 7 Service de dermatologie, Centre Hospitalier Universitaire de Bordeaux, Bordeaux, France; 8 Service de chirurgie plastique, reconstructrice et esthétique -brulés- chirurgie de la main, Centre Hospitalier Universitaire de Bordeaux, Bordeaux, France; Institut de Pharmacologie et de Biologie Structurale, FRANCE

## Abstract

**Background:**

Nontuberculous mycobacteria (NTM) are environmental organisms associated with a range of infections. Reports of NTM epidemiology are mainly focused on pulmonary infections and isolations, and extrapulmonary infections are less frequently described.

**Methods:**

We conducted a retrospective study of NTM infections at the Bordeaux University Hospital, France, between January 2002 and December 2013. We used the microbiologic component of the American Thoracic Society/Infectious Diseases Society of America's pulmonary NTM disease criteria to define cases of pulmonary NTM, and patients with isolates from a normally sterile site were classified as having extrapulmonary disease.

**Results:**

In our setting, 170 patients were included. Pulmonary cases predominated (54.1%), followed by skin and soft tissue infections (22.9%), disseminated cases (10.6%), lymphadenitis (7.7%), bone and joint infections (2.9%) and the remaining 1.8% catheter-related infections. Overall, 16 NTM species were isolated. *Mycobacterium avium* (31.8%) and *M*. *intracellulare* (20%) were the most common species identified, followed by *M*. *marinum* (13.5%), *M*. *kansasii* (10.6%), *M*. *xenopi* (9.4%), rapidly growing mycobacteria (9.4%) and other slowly growing mycobacteria (5.3%). In general, NTM isolates were largely prevalent in people older than 50 (62.4%); patients aged 1–10 year-old exclusively yielded *M*. *avium* from lymph nodes, almost cases having being diagnosed after 2007. Among the 121 patients with complete follow-up, 78 (64.5%), 24 (19.8%), and 19 (15.7%) were cured, experienced relapse, or died, respectively.

**Conclusion:**

In our study, extrapulmonary NTM infections represented almost half of cases, consisting mainly in skin and soft tissue infections. The increase lymphadenitis cases in children after 2007 could be linked to the cessation of mandatory BCG vaccination in France. We observed similar cure rates (64%) between pulmonary and extrapulmonary infections.

## Introduction

Nontuberculous mycobacteria (NTM) are environmental organisms found in soil and water throughout the world [1]. Unlike *Mycobacterium tuberculosis*, which is a genuine pathogen, the largely environmental NTM have been often associated with conditions of host impaired immunity, such as primary immunodeficiency, Human Immunodeficiency Virus (HIV) infection or the use of immunosuppressive medication. Increasingly, NTM are isolated in immunocompetent individuals, with or without pre-existing structural lung damage. NTM are a group of over 150 species, but, due to recent advancement of molecular techniques, novel species are being described [2]. They are considered opportunistic pathogens, and several species are associated with human disease. Pulmonary infections are the most frequent, followed by skin/soft tissue infections, lymphadenitis in children, or disseminated infections in severely immunocompromised patients. NTM are an important cause of morbidity and mortality, often on the form of progressive lung disease.

Although significant differences in geographic of NTM have been observed [3], *Mycobacterium avium* and *Mycobacterium intracellulare*, species belonging to *Mycobacterium avium* complex (MAC), are the most frequently reported. Other important human NTM pathogens included slowly growing mycobacteria (SGM), such as *Mycobacterium kansasii*, *Mycobacterium xenopi*, *Mycobacterium malmoense*, *Mycobacterium marinum* and rapidly growing mycobacteria (RGM) such as *Mycobacterium abscessus*, *Mycobacterium chelonae* and *Mycobacterium fortuitum* [4].

NTM infections are rare (1.0 to 1.8 cases per 100 000 persons) [4], even though their numbers increased during the last 20 years [5]. The diagnosis of mycobacterial infections remains difficult, relying on clinical, bacteriological and radiological criteria, especially for pulmonary disease [4].

We conducted an epidemiological and clinical retrospective study on all NTM infections diagnosed at the Bordeaux University Hospital (France) over a 12-year period. We aimed to describe microbiological and clinical characteristics, and patient outcomes.

## Methods

The database of the bacteriological laboratory at the Bordeaux University Hospital was used to identify patients with isolation of NTM between January 2002 and December 2013. Only patients followed up at the Bordeaux University Hospital were included in the study. Demographic (gender, age) and clinical data (site of infection, underlying disease, impaired immunity, symptoms, radiographic results, treatment and outcome) were obtained from clinical records.

Diagnosis criteria for NTM lung disease were those of the American Thoracic Society (ATS)/Infectious Diseases Society of America (IDSA) recommendations [4). Patients with cystic fibrosis were excluded. Radiographic abnormalities (nodular, infiltrative or cavitary) were analyzed by chest radiography and/or CT scanning.

We categorized extrapulmonary NTM disease cases as: skin and soft tissue; disseminated; lymphadenitis; bone and joint or catheter-related infections. We defined a case of skin and soft-tissue disease as one or more isolates from tissue samples labeled as “furuncles”, “abscesses”, “skin”, “wound”, or “synovial fluid”. We defined a disseminated case as NTM isolation from blood, bone marrow or other sterile site body fluids such as spinal site. Case of lymphadenitis was defined as isolation of NTM from a lymph node biopsy or aspirate. The category of bone and joint was used for positive bone biopsy or joint fluid cultures. Catheter-related infection was defined as isolation of NTM from normally sterile material.

Microscopic examination of the samples was performed by Zielh-Neelsen staining, and specimens were inoculated onto a Löwenstein-Jensen and a Coletsos sold media (Bio-Rad, Marnes La coquette, France) and onto a liquid culture media using the Bactec MGIT 960 mycobacterial detection systems (Becton Dickinson, USA). NTM identification was based on molecular characterization. According to morphology of the bacilli on Ziehl-Neelsen, the appearance and pigmentation of colonies on solid media and the growth rate, we first used hybridization with DNA probes (AccuProbe^®^) available for *M*. *avium* complex, *M*. *avium*, *M*. *intracellulare*, *M*. *gordonae* or *M*. *kansasii* when we suspected one this species. If the result was negative or if we suspected another species, we used the GenoType Mycobacterium CM/AS line probe assays (Hain Lifesciences) as recommended by the manufacturer. If all these tests were inconclusive, 16S DNA sequencing was realized.

Statistical analysis was performed using the chi-square test (or Fischer’s exact test when appropriate) for categorical variables and the Krustal-Wallis test for continuous variables. A p-value of <0.05 was considered as significant.

This study was conducted according to the principles expressed in the Declaration of Helsinki. The study was conducted in accordance with the guidelines of the “Direction de la Recherche Clinique et de l’Innovation", the research board of Bordeaux University hospital, Bordeaux, France. Informed consent was waived because this study only used the data gained from clinical practice. All patient data were anonymously reported, with no possibility of connecting the isolates and specimens to individual patients.

## Results

A total of 198 patients were retrospectively selected. Twenty-eight were excluded because of the lack clinical data. Overall, 170 patients were included in the study. We identified 92 (54.1%) and 78 (45.9%) patients with pulmonary and extrapulmonary infections, respectively (Table 1). There were 104 men (61.2%) and 66 women (38.8%) with a median age of 55.5 years [1–92]. Patients with pulmonary infections were significantly older than those with extrapulmonary infections (61.5 vs 52 years, p = 0.001). Seventy-six patients (45.2%) had immunodeficiency of whom 36 (21.4%) were HIV-positive. During the study period 16 different mycobacterial species were identified (Table 2). No patient was infected by more than one NTM species.

**Table 1:** Characteristics of the 170 subjects included in the study.

Characteristics	Pulmonary N = 92	Extrapulmonary N = 78	p-value
Median age, years (range)	61.5 (6–92)	52 (1–90)	0.001
Sex male/female	53/39	51/27	0.3
Impaired immunity^a^, no. (%)	40^b^ (44.4)	36 (46.2)	0.82
HIV-infection	16 (17.8)	20 (25.6)	0.22
Neoplasia	19 (21.1)	10 (12.8)	0.16
Immune defect	3 (3.2)	2 (2.6)	1
Immunosuppressive medication	5 (5.6)	4 (5.1)	1

^a^Patients can belong to more than of these categories.

^b^Data were not available for two patients.

HIV: Human Immunodeficiency Virus

**Table 2:** Species and site of infection of NTM isolated at the Bordeaux University Hospital, France, between 2002 and 2013 (n = 170).

NTM species	Number of isolates						Total
	Pulmonary	Skin/soft tissue	Disseminated	Lymphadenitis	Bone/joint	Catheter	
*M*. *avium*	27	2	13	12	-	-	**54**
*M*. *intracellulare*	27	5	1	-	1	-	**34**
*M*. *marinum*	0	23	-	-	-	-	**23**
*M*. *kansasii*	16	-	-	1	1	-	**18**
*M*. *xenopi*	15	1	-	-	-	-	**16**
*M*. *chelonae*	0	5	1	-	2	2	**10**
*M*. *abscessus*	2	1	-	-	1	-	**4**
*M*. *simiae*	1	-	2	-	-	-	**3**
*M*. *fortuitum*	1	-	-	-	-	-	**1**
*M*. *scrofulaceum*	1	-	-	-	-	-	**1**
*M*. *terrae*	-	1	-	-	-	-	**1**
*M*. *szulgaï*	-	1	-	-	-	-	**1**
*M*. *genavense*	-	-	1	-	-	-	**1**
*M*. *interjectum*	1	-	-	-	-	-	**1**
*M*. *gordonae*	1	-	-	-	-	-	**1**
*M*. *peregrinum*	**-**	**-**	**-**	**-**	**-**	1	**1**
**Total**	**92**	**39**	**18**	**13**	**5**	**3**	**170**

### Pulmonary infections

The median age of infected patients was 61.5 years [6–92] (Table 1). NTM isolates were prevalent in people older than 60 years (n = 49, 53.3%). Structural lung damage predisposing to NTM pulmonary infection was reported in 42 cases (13 patients had chronic obstructive pulmonary disease, 16 had bronchectiasis, 6 had emphysema, 11 had a history of pulmonary tuberculosis infection and 5 had other causes of chronic pulmonary disease). Thirty five patients (38%) reported smoking actively at the time of their infection. Interestingly, 52.2% (48/92) and 54.3% (50/92) of the patients have no detected underlying lung disease or immunodeficiency, respectively.

A median of two specimens [1–8] were obtained (52 bronchial aspirates, 65 bronchoalveolar lavages, 95 sputum, 31 gastric aspirates, 10 lung biopsies, 1 bronchial biopsy) with a median of two positive specimens [1–8] (S1 Table). Twenty-nine patients had one or more specimens with positive smear. Histological examination, realized in 16 cases, revealed epithelio-gigantocellular granuloma with or without caseous necrosis for ten patients (S1 Table). Mainly all NTM cultured from pulmonary samples were SGM (97%; 89/92): the most common species were *M*. *avium* (27 isolates) and *M*. *intracellulare* (27 isolates) followed by *M*. *kansasii* (16 isolates) and *M*. *xenopi* (15 isolates), while *M*. *scrofulaceum*, *M*. *simiae*, *M*. *gordonae* and *M*. *interjectum* were only isolated once (Table 2). A case of *M*. *gordonae* pulmonary infection was diagnosed in a patient with pulmonary symptoms and cavitations on CT scan; two lung biopsies and two gastric aspirates were culture-positive, one gastric aspirate having a positive-smear, and histological examination of lung biopsies was contributive (S1 Table). Among the three (3%) infections caused by RGM, two were due to *M*. *abscessus* and one to *M*. *fortuitum*.

The main characteristics of patients with *M*. *avium*, *M*. *intracellulare*, *M*. *xenopi* or *M*. *kansasii* infections are presented in Table 3. Median age was >60 years for *M*. *avium* and *M*. *intracellulare*, whereas it was 51 and 52 years for *M*. *kansasii* and *M*. *xenopi*, respectively (p = 0.45). Male patients tended to be more frequently infected by *M*. *kansasii* (81.2%, 13/16) (p = 0.07). Pre-existing pulmonary disease was found in 46.2% (12/26) of the subjects infected with *M*. *avium*, 59.3% (16/27) with *M*. *intracellulare* and 26.7% (4/15) with *M*. *xenopi* or *M*. *kansasii* (p = 0.11). Chronic obstructive pulmonary disease and bronchectiasis were the most frequent ones for *M*. *avium* and *M*. *intracellulare* pulmonary disease. A significant higher rate of bronchectiasis was observed in patients infected with *M*. *intracellulare* (33.3%, 9/26) than in those infected with *M*. *avium* (15.4%, 4/26), *M*. *kansasii* (6.7%, 1/15) or *M*. *xenopi* (0%) (p = 0.03). Impaired immunity was more found in subjects with *M*. *avium* (53.8%, 14/26), *M*. *intracellulare* (44.4%, 12/27) or *M*. *xenopi* (53.3%, 8/15) than those infected with *M*. *kansasii* (13.3%, 2/15), though this observation did not reach statistical significance (p = 0.06). HIV-positive status was significantly more frequent in subjects infected with *M*. *xenopi* (33.3%, 5/15) than in those with *M*. *avium* (26.9%, 7/26) or *M*. *intracellulare* (11.1%, 3/27) (p = 0.03). Patients with *M*. *kansasii* pulmonary disease were HIV-negative. Constitutional symptoms, mainly fever and impaired clinical status were not common: 48.8% (13/27) for *M*. *avium*, 37% (10/27) for *M*. *intracellulare*, 40% (6/15) for *M*. *xenopi* and 50% (8/16) for *M*. *kansasii* pulmonary disease (p = 0.79). However, most patients presented pulmonary symptoms, such as cough and dyspnoea, with no significant difference between patients infected by different species (p = 0.86). Haemotypsis was the only clinical sign in two patients with *M*. *intracellulare* pulmonary disease. Nodules were the predominant radiological pattern in subjects infected by *M*. *avium* (64.3%, 9/14), *M*. *intracellulare* (50%, 11/22) and *M*. *kansasii* (50%, 6/12). Similar rate (35.7%, 5/14) of cavitations and nodules was observed in patients infected by *M*. *xenopi*. Whatever the species involved, the majority of patients received antibiotic treatment, mainly with three drugs (clarithromycin, ethambutol and rifabutin). The median duration of the treatment ranged from 10 months [2–36] for *M*. *avium* and 10.5 months [5–16] for *M*. *kansasii* to 12 months for *M*. *intracellulare* [4–36] and *M*. *xenopi* [2–36]. Among the 20 patients treated for *M*. *avium* pulmonary disease, 13 were considered as cured, four relapsed, one was lost to follow-up, and two died. Similarly, among the 20 subjects treated for *M*. *intracellulare* pulmonary infection, 12 cured, two relapsed, four were lost to follow up and two died. Among patients treated for *M*. *xenopi*, five cured whereas five relapsed; two were lost to follow-up and two died. For *M*. *kansasii*, eight patients were cured after treatment, three were lost to follow-up and one died; none relapsed.

**Table 3:** Comparison of the characteristics of the patients with *M*. *avium*, *M*. *intracellulare*, *M*. *xenopi* or *M*. *kansasii* pulmonary disease included the study.

	Number of cases of NTM pulmonary disease				p-value
	*M*. *avium*	*M*. *intracellulare*	*M*. *xenopi*	*M*. *kansasii*	
**Number of subjects**	27	27	15	16	
**Median age, years (range)**	62 (31–92)	68 (6–92)	52 (40–83)	51 (18–89)	0.45
**Sex male/female**	17/10	11/16	9/6	13/3	0.07
**Pre-existing pulmonary disease**^a^, n(%)	**12**^b^ **(46.1)**	**16 (59.3)**	**4 (26.7)**	**4**^b^ **(26.7)**	0.11
COPD, n(%)	4 (15.4)	5 (18.5)	2 (13.3)	2 (13.3)	1
Bronchiectasis, n(%)	4 (15.4)	9 (33.3)	0	1 (6.7)	0.03
Tuberculosis in the past, n(%)	2 (7.7)	2 (7.4)	3 (20)	2 (13.3)	0.60
Emphysema, n(%)	1 (3.8)	3 (11.1)	0	0	0.52
Other, n(%)	2 (7.7)	1 (3.7)	1 (6.7)	1 (6.7)	1
**Impaired immunity**^a^, n(%)	**14**^b^ **(53.8)**	**12 (44.4)**	**8 (53.3)**	**2**^b^ **(13.3)**	0.06
HIV-infection, n(%)	7 (26.9)	3 (11.1)	5 (33.3)	0	0.03
Neoplasia, n(%)	6 (23.1)	7 (25.9)	2 (13.3)	2 (13.3)	0.71
Immune defect, n(%)	1 (3.8)	2 (7.4)	0	0	0.77
Immunosuppressive medication, n(%)	2 (7.7)	0	1 (6.7)	0	0.33
**General clinical symptoms**^a^, n(%)	**13 (48.1)**	**10 (37)**	**6 (40)**	**8 (50)**	0.79
Fever, night sweats, n(%)	9 (33.3)	5 (18.5)	3 (20)	1 (6.2)	0.21
Weight loss, n(%)	0	1 (3.7)	0	0	1
Asthenia, n(%)	2 (7.4)	2 (7.4)	1 (6.7)	0	0.80
Impaired clinical status, n(%)	5 (18.5)	5 (18.5)	4 (26.7)	7 (43.8)	0.26
**Pulmonary symptoms**^a^, n(%)	**18 (66.7)**	**19 (70.4)**	**12 (80)**	**11 (68.8)**	0.86
Cough, n(%)	13 (48.1)	14 (51.8)	8 (53.3)	5 (31.3)	0.55
Dyspnoea, n(%)	10 (37)	7 (25.9)	8 (53.3)	4 (25)	0.28
Haemotypsis, n(%)	1 (3.7)	4 (14.8)	1 (6.7)	1 (6.3)	0.55
**Radiographic abnormalities**^a^, n(%)	**14**^c^ (100)	**22**^c^ (100)	**14**^c^ (100)	**12**^c^ (100)	1
Infiltrates, n(%)	3 (21.4)	3 (13.6)	3 (21.4)	3 (25)	0.84
Nodules, n(%)	9 (64.3)	11 (50)	5 (35.7)	6 (50)	0.54
Cavitation, n(%)	3 (21.4)	4 (18.2)	5 (35.7)	2 (16.7)	0.67
Nonspecific, n(%)	2 (14.3)	6 (27.3)	2 (14.3)	1 (8.3)	0.61
**Treatment**, n(%)	**20 (74)**	**20 (74)**	**14 (93.3)**	**12 (75)**	0.47
Surgery, n(%)	1 (3.7)	0	0	0	-
Antibiotherapy, n(%)	19 (70.3)	20 (74)	14 (93.3)	12 (75)	0.37

^a^Patients can belong to more than one of these categories.

^b^Data were not available for one patient.

^c^No radiological data were available for 13 patients infected with *M*. *avium*, five with *M*. *intracellulare*, one with *M*. *xenopi* and four with *M*. *kansasii*.

COPD: chronic obstructive pulmonary disease.

### Extrapulmonary infections

We identified 78 patients with extrapulmonary manifestations (45.9%). Skin and soft-tissue infections were the most common, accounting for 50% (n = 39) of these manifestations in the 12-years period. Almost all specimens received for microbial diagnosis were skin biopsies, the remaining being synovial biopsies (S2 Table). Smear was positive in only three cases. Most infections were due to *M*. *marinum* (59%, 23/39), followed by RGM (15.4%, 6/39), *M*. *intracellulare* (12.8%, 5/39), *M*. *avium* (5.1%, 2/39), and *M*. *terrae*, *M*. *xenopi*, *M*. *szulgaï* isolated once. Histological examination, realized in 31 cases, showed granulomatous inflammation with or without caseous necrosis in 21 cases (S2 Table). Among the 23 patients with *M*. *marinum*, infection was limited to a skin disease on the limbs for 18 patients but spread to deeper structures, resulting in tenosynovitis, for five patients. Overall, 17 received treatment, one did not after medical decision and no information about treatment was available for five patients. Monotherapy was prescribed for 11 patients, mostly with clarithromycin (n = 9), azithromycin (n = 1) and doxycycline (n = 1). Five patients received two antibiotics and one was treated with three drugs. Median duration of antibiotherapy was 3 months [1–12]. Surgery was performed only for patients with tenosynovytis, in association to bi- or- tri-antibiotherapy. Clinical course, obtained for 11 treated patients, revealed cure for 10 of them and relapse for only one. Among the 10 patients with SGM other than *M*. *marinum* skin and soft-tissue infections, five had tenosynovitis (3 with *M*. *intracellulare*, one with *M*. *terrae* and one with *M*. *xenopi*); the five remaining, of whom two were immunosuppressed, presented nodular lesions. Nine patients received antibiotics (from one to four drugs), with a median duration of eight months [6–30]; clinical cure was observed for four patients, relapse in three cases and two were lost to follow-up. The tenosynovitis with *M*. *terrae* was diagnosed in a farmer, first in 2004 after tenosynovectomy, and then in 2008 and 2010 because of relapse requiring new surgery. Among the six patients with infection caused by a RGM, three were immunocompetent and skin lesions (nodules, cutaneous necrosis) appeared after piercing or cosmetic surgery; the three others received immunosuppressive treatments and presented multiples nodules on the upper or lower limbs. All, except one, were treated with one or two antibiotics, with a median duration of five months [3–6] and experienced clinical cure.

Disseminated cases were 10.6% of the total NTM cases. All patients were immunosuppressed, of whom 83.3% (15/18) were HIV-positive. The median number of specimens received per patient was 5 [1–9] with a median of two positive specimens [1–8]. Most infections were due to *M*. *avium* (72.2%, 13/18) (S2 Table). The disseminated infection with *M*. *chelonae* was reported in a man who had a tracheotomy post-radiation since seven years and who was hospitalized for pace-maker exteriorization following fall; all specimens (bronchial aspirates, sample of the lodge of the pace-maker and blood culture) grew with *M*. *chelonae*. Fourteen patients received a combination of two or three antibiotics from one to 22 months (median: 7.5 months). Five were cured, two relapsed, three were lost to follow-up and four died. Three patients were not treated because death occurred shortly after the discovery of the MNT and one patient was lost to follow-up. Deaths were attributed to the NTM infection only in one case (*M*. *chelonae* disseminated infection), to the underlying disease in seven cases and to another cause in one case.

Thirteen cases of lymphadenitis were identified (S2 Table). They mostly occurred in children (n = 8) aged between 1 and 4 years, of whom six were girls. Regarding BCG vaccination status, six children were not vaccinated and status was unrecorded for two. Locations were submandibular (n = 6) or parotid (n = 1); one patient had two distinct lesions (submandibular and preauricular). None had constitutional symptoms. At diagnosis, painless and firm lymphadenopathy was observed in 72% of cases, skin changes associated with violaceus coloration in 14% and cutaneous fistulae in 14%. Direct smear was positive in only two cases and histological examination was contributive in four cases. All cases were due to *M*. *avium*. Five children were treated by surgery and antibiotics, one exclusively by surgery, one by antibiotherapy and one received no treatment; two patients presented relapse. Among the five cases that occurred in adults, four were HIV-positive. Location was mainly cervical (60%). Direct smear was positive in only one case and histological results were not specific. *M*. *avium* was identified in four subjects and *M*. *kansasii* in one. Four patients were treated with a combination of two or three antibiotics during a median duration of 11 months [9–21]; two patients died because of underlying disease, one relapsed, one observed clinical cure and one was lost to follow-up.

Bone and joint infections were diagnosed in five cases (2.9% of all NTM cases) (S2 Table). All patients were immunocompetent, except one who was HIV-positive. Direct smear was negative in all cases. Three infections were due to RGM: a septic arthritis with *M*. *abscessus* after needle injection of corticosteroids, a bone infection of a knee with *M*. *chelonae* after a ligamentoplasty and bone infection of a foot with *M*. *chelonae*. These patients were treated during 4 or 7 months and cure was obtained for all of them. A prosthetic joint infection was due to *M*. *intracellulare* and successfully treated after one year of antibiotherapy. The last case, a bone infection of the elbow caused by *M*. *kansasii*, relapsed despite appropriate treatment.

Two port infections due to *M*. *chelonae* were identified, and data obtained for one revealed cured after three months of antibiotherapy. One case of cardiac device-related infective endocarditis due to *M*. *peregrinum* was recorded; the patient was successfully treated after surgery and three years of antibiotherapy.

For the treatment of NTM extrapulmonary infections, the three most used antibiotics were clarithromycin (80.6%, 50/62), rifabutin (41.9%, 26/62) and ethambutol (38.7%, 24/62).

## Discussion

Similar to other reports, NTM isolations from pulmonary sites occurred most frequently [6, 7]. Pre-existing chronic lung diseases, especially chronic obstructive pulmonary disease, asthma and bronchiectasis, are the main risk factors for NTM pulmonary infection [4]. However, consistent with our findings, a considerable proportion of patients with this disease have no detected underlying lung disease or immunodeficiency [8].

In the present study, the species distribution of NTM isolated in pulmonary disease was close to that reported earlier in France [9] and more recently in an inventory study of NTM in the European Union, which included France [3]. MAC was the commonest group of cultured organisms, as reported worldwide [3]. While studies showed that *M*. *avium* was the predominant subspecies recovered from human biospecimens [3, 10], we found a similar number of *M*. *avium* and *M*. *intracellulare* isolates in our cohort. *M*. *xenopi*, followed by *M*. *kansasii* and *M*. *malmoense*, is more frequently encountered than is *M*. *abscessus* in non-cystic fibrosis patients [3].

In Europe, the manifestations of the MAC pulmonary infection vary in the majority of cases. In the Netherlands, Van Ingen et al. reported that patients with MAC pulmonary disease more often had cavitary than nodular bronchiectatic disease [11]. In contrast, in France, among patients with MAC pulmonary infection, there were lower rates of fibrocavitary than of nodular bronchectatic disease [9, 10], which is similar to the results of recent surveys in the USA and South Asia [12–15] and to our results. It is of interest to note that patients infected with *M*.*avium* or *M*. *intracellulare* have similar radiological characteristics in our study. It also appears that infections with *M*. *kansasii*, *M*. *malmoense* or *M*. *xenopi* were more frequently associated with fibrocavitary disease [8].

Similarly to previous reports [6], we didn’t found any association between NTM pulmonary infection and gender, although *M*. *kansasii* appeared to be more common in men (81.2%, 13/16). In contrast, some authors described highest prevalence of MAC infection in women [9].

Previous investigations described increase rates of NTM disease in the elderly [6, 7, 16]. We found that the age distribution differed among isolation sites. We reported a rise isolation prevalence of pulmonary disease in the oldest (60 years and over] group. For extrapulmonary NTM infections, the highest rates occurred in the “40–59 years” and “60 years and older”.

Interestingly, in our cohort, although pulmonary disease was predominant, we observed a high rate of extrapulmonary manifestations (45.9%). This is more than that described in literature, with prevalence around 20–25% [6, 7, 17]. Among them, skin and soft tissue infections were the most encountered. Overall, 70% of cutaneous isolates were *M*. *marinum* or RGM, which has also been noted by others [18]. *M*. *marinum* was isolated exclusively from skin samples, reflecting the ability of the mycobacterial species to infect and localize in different body sites [19]. *M*. *marinum* human infections are generally cutaneous, although can become deeper in some cases resulting in tenosynovitis, arthritis and osteomyelitis [20]. Antimicrobial susceptibility testing is not recommended as *M*. *marinum* is usually susceptible to antimicrobials used for treatment, including rifampicin, ethambutol, doxycycline–minocycline, co-trimoxazole and clarithromycin [21]. Testing is however recommended for those that fail to respond to treatment after several months and with positive cultures. As previously described [22], the median duration of antibiotic therapy was three months in our study and recovery was obtained in 90% of the cases. Because *M*. *marinum* infection is usually localized, single therapy is widely used. Aubry et al. reported that the duration of antibiotics was significantly longer for patients with deeper structure infections than for patients with infections limited to the skin and soft tissue [22], and that surgery was indeed associated with deep structure infection.

As for pulmonary infections, MAC was the commonest group of cultured organisms from extrapulmonary sites [16], mainly responsible of disseminated cases and lymphadenitis. Disseminated mycobacterial infections developed in immunocompromised people, most being HIV-positive (83.3% in our work) [19]. Inside the MAC, *M*. *avium* was isolated in all cases, except one for which *M*. *intracellulare* was identified, which is consistent with the current literature [19, 23].

In our study, lymphadenitis is the clinical manifestation of NTM infection of children. It occurred in immunocompetent children less than 5 years old, which is consistent with other reports [23]. *M*. *avium*, the sole species identified in the eight cases of our cohort, is the main cause of these infections [24]. *M*. *malmoense* is frequently described in the United Kingdom and Sweden, *M*. *haemophilum* in Israel and The Netherlands [24], and *M*. *lentiflavum* in Spain [24, 25]. It is of interest to note that none of the eight children was vaccinated with the BCG and that all cases were diagnosed after 2007, year of the cessation of compulsory BCG vaccination in France, suggesting possible cross protection by BCG to mycobacteriosis.

Our therapeutic approach was in accordance with IDSA guidelines [4]. Duration of treatment varies according to the species and localization. Treatment duration of 12 months after sputum culture conversion is generally recommended for most NTM pulmonary disease [4]. The cornerstones of most anti-NTM drug regimens are macrolides, but there are exceptions such as *M*. *kansasii*.

In our cohort, deaths of patients with NTM pulmonary disease were not attributed to NTM infection, but to underlying disease (six cases) or another cause (three cases). This result is of interest as recent follow-up studies have indicated that pulmonary NTM findings carry a high mortality rate, attributed to the NTM infection, ranging from 24 to 69% within 5 years, [26–32]. The mortality among patients with atypical mycobacteria differs per species. In a population-based study from Denmark, *M*. *xenopi* was associated to poorer prognosis than MAC [27]. In accordance with these results, in a retrospective study from Helsinki, pulmonary MAC patients had significantly lower risk of death as compared to patients with pulmonary infection of other SGM and as compared to patients with pulmonary RGM [32]. The treatment success rate is higher (70–85%) in patients with non-cavitary nodular bronchiectasis MAC lung disease than in those with cavitary MAC lung disease [33]. The result obtained in our study could be explained by the high number of treated patients (72/92, 78.3%), the use of recommended regimens for MAC and *M*. *kansasii*, and a highest number of patients with nodular and infiltrates presentation than that fibrocavitary form.

The use of more recent drugs such as tigecycline may have allowed a better control of RGM infection. In our study, tigecycline was included in the initial treatment regimen in four patients with extra-pulmonary infection, in combination to one or more antibiotics, and complete resolution was observed after treatment courses of four to seven months. Tigecycline has consistently demonstrated excellent activity *in vitro* against clinical isolates of both tetracycline susceptible and tetracycline-resistant *M*. *abscessus* and *M*. *chelonae*, but few reports of clinical experience with tigecycline for the treatment of RGM infections are found in the medical literature. Recently, Wallace et al. reported the efficacy of tigecycline as part of an antibiotic regimen for salvage treatment of *M*. *abscessus* and *M*. *chelonae* infections in a large cohort of 52 patients [34].

This study has several limitations. First, we did not collect data outside of hospital. Second, incomplete reporting could undermine the accuracy of our estimates. Finally, due to the retrospective nature of the study accounts for the lack of information is missing regarding patient follow-up in 28% of the cases or sometimes in the justification for the absence treatment.

In conclusion, the present study, although not representing a population-based investigation, provides a snapshot of the prevalent NTM species in our setting. Extrapulmonary NTM infections represented almost half of cases, consisting mainly in skin and soft tissue infections. Clinicians should be aware of the increase lymphadenitis cases in children observed after 2007, which could be linked to the cessation of compulsory BCG vaccination in France. We observed similar cure rates (64%) between pulmonary and extrapulmonary infections.

## Supporting Information

S1 Table Microbiological and histological characteristics for the 92 patients with NTM pulmonary infection. ^a^ When multiple specimens were received, the number and the origin of each of them are indicated in parentheses. ^b^ The number and the origin of the positive specimens are indicated in parentheses. BAL: bronchoalveolar lavage; NR: not realized.
(DOCX)

S2 Table Microbiological and histological characteristics for the 78 patients with NTM extrapulmonary infection. ^a^ When multiple specimens were received, the number and the origin of each of them are indicated in parentheses. ^b^ The number and the origin of the positive specimens are indicated in parentheses. BAL: bronchoalveolar lavage; NR: not realized.
(DOCX)
