# Supplementary material for: Nontuberculous Mycobacterial Infections in a French Hospital: A 12-Year Retrospective Study
Source: PLoS One. 2016 Dec 13;11(12):e0168290. doi: 10.1371/journal.pone.0168290 (PMC5154556; doi:10.1371/journal.pone.0168290)
Supplement: S1 Table — a When multiple specimens were received, the number and the origin of each of them are indicated in parentheses. b The number and the origin of the positive specimens are indicated in parentheses. BAL: bronchoalveolar lavage; NR: not realized. (DOCX) [file pone.0168290.s001.docx]

**S1 Table. Microbiological and histological characteristics for the 92 patients with NTM pulmonary infection.**

| **Patient number** | **Sampling date** | **Number of specimens received** | **Specimens origin**^a^ | **Number of specimens**  **with positive culture**^b^ | **Number of specimens with positive smear and positive culture**^b^ | **Isolation date** | **Species** | **Histological results** |
| --- | --- | --- | --- | --- | --- | --- | --- | --- |
| 1 | 10/09/2004 | 2 | bronchial aspirate (1), BAL (1) | 1 (bronchial aspirate) | 0 | 05/11/2004 | *M. avium* | NR |
| 2 | 10/06/2009 | 1 | BAL | 1 | 0 | 22/06/2009 | *M. avium* | NR |
| 3 | 09/06/2008 | 2 | bronchial aspirate (1), BAL (1) | 1 (bronchial aspirate) | 0 | 16/06/2008 | *M. avium* | NR |
| 4 | 17/02/2010 | 5 | bronchial aspirate (1), BAL (1), gastric aspirate (3) | 1 (bronchial aspirate) | 0 | 01/03/2010 | *M. avium* | NR |
| 5 | 19/10/2010 | 1 | BAL | 1 | 0 | 25/10/2010 | *M. avium* | NR |
| 6 | 22/12/2008 | 1 | BAL | 1 | 0 | 05/01/2009 | *M. avium* | NR |
| 7 | 05/01/2006 | 1 | BAL | 1 | 0 | 30/01/2006 | *M. avium* | NR |
| 8 | 12/04/2007 | 1 | BAL | 1 | 0 | 26/04/2007 | *M. avium* | NR |
| 9 | 08/06/2010 | 1 | lung biopsy | 1 | 1 | 16/06/2010 | *M. avium* | granuloma with caseous necrosis |
| 10 | 03/11/2003 | 5 | bronchial aspirate (1), BAL (1), sputum (2), bronchial biopsy (1) | 4 (bronchial aspirate, sputum (2), bronchial biopsy) | 0 | 12/11/2003 | *M. avium* | not specific |
| 11 | 18/04/2011 | 7 | lung biopsy (1), sputum (3), gastric aspirate (3) | 1  (lung biopsy) | 0 | 04/05/2011 | *M. avium* | epithelio-gigantocellular granuloma with caseous necrosis |
| 12 | 23/05/2009 | 3 | bronchial aspirate (1), BAL (1), sputum (1) | 2 (bronchial aspirate, sputum) | 0 | 02/06/2009 | *M. avium* | NR |
| 13 | 27/10/2004 | 3 | bronchial aspirate (1), BAL (1), sputum (1) | 1  (bronchial aspirate) | 0 | 03/11/2004 | *M. avium* | NR |
| 14 | 28/03/2013 | 1 | BAL | 1 | 0 | 08/04/2013 | *M. intracellulare* | NR |
| 15 | 02/01/2008 | 3 | sputum | 3 | 0 | 07/01/2008 | *M. fortuitum* | NR |
| 16 | 10/06/2006 | 5 | lung biopsy (2), gastric aspirate (3) | 4 (lung biopsy (2), gastric aspirate (2)) | 1 (gastric aspirate) | 17/06/2006 | *M. gordonae* | epithelio-gigantocellular granuloma with caseous necrosis |
| 17 | 07/11/2012 | 7 | bronchial aspirate (1), BAL (1), sputum (5) | 3 (bronchial aspirate, sputum (2)) | 0 | 23/11/2012 | *M. interjectum* | NR |
| 18 | 14/03/2012 | 5 | BAL (1), sputum (1), gastric aspirate (3) | 5 | 2 (gastric aspirate, sputum) | 02/04/2012 | *M. intracellulare* | NR |
| 19 | 21/02/2009 | 3 | gastric aspirate | 2 | 0 | 10/03/2009 | *M. intracellulare* | NR |
| 20 | 02/02/2013 | 2 | sputum (1), BAL (1) | 1 (BAL) | 0 | 18/02/2013 | *M. intracellulare* | NR |
| 21 | 20/12/2006 | 2 | bronchial aspirate (1), sputum (1) | 2 | 1 (bronchial aspirate) | 26/12/2006 | *M. intracellulare* | NR |
| 22 | 15/01/2010 | 2 | bronchial aspirate (1), BAL (1) | 1 | 0 | 25/01/2010 | *M. intracellulare* | NR |
| 23 | 13/07/2010 | 3 | bronchial aspirate (1), BAL (1), sputum (1) | 3 | 0 | 16/08/2010 | *M. intracellulare* | NR |
| 24 | 07/10/2008 | 7 | BAL (1), sputum (3), gastric aspirate (3) | 2  (gastric aspirate) | 1 (gastric aspirate) | 17/10/2008 | *M. intracellulare* | NR |
| 25 | 13/11/2012 | 5 | bronchial aspirate (1), BAL (1), sputum (3) | 2 (bronchial aspirate, BAL) | 0 | 23/11/2012 | *M. intracellulare* | epithelio-gigantocellular granuloma without caseous necrosis |
| 26 | 30/05/2011 | 3 | sputum | 2 | 0 | 15/07/2011 | *M. intracellulare* | NR |
| 27 | 28/06/2012 | 2 | bronchial aspirate (1), BAL (1) | 2 | 0 | 04/07/2012 | *M. intracellulare* | NR |
| 28 | 29/07/2009 | 2 | bronchial aspirate (1), BAL (1) | 1 (BAL) | 0 | 18/08/2009 | *M. intracellulare* | NR |
| 29 | 02/07/2002 | 3 | bronchial aspirate (1), BAL (1), sputum (1) | 2 (bronchial aspirate, sputum) | 0 | 15/07/2002 | *M. kansasii* | NR |
| 30 | 27/10/2007 | 1 | pleural fluid | 1 | 0 | 05/12/2007 | *M. kansasii* | NR |
| 31 | 25/03/2009 | 3 | bronchial aspirate (1), sputum (2) | 3 | 3 | 30/03/2009 | *M. kansasii* | NR |
| 32 | 03/10/2012 | 6 | bronchial aspirate (1), BAL (1), lung biopsy (1), gastric aspirate (3) | 3 (bronchial aspirate, BAL, lung biopsy) | 1 (lung biopsy) | 10/10/2012 | *M. kansasii* | NR |
| 33 | 24/06/2009 | 2 | bronchial aspirate (1), BAL (1) | 2 | 0 | 06/07/2009 | *M. kansasii* | NR |
| 34 | 11/04/2005 | 6 | bronchial aspirate (1), BAL (1), sputum (3), gastric aspirate (1) | 3 (bronchial aspirate, BAL, sputum) | 3 | 18/04/2005 | *M. kansasii* | NR |
| 35 | 09/09/2004 | 3 | bronchial aspirate (1), BAL (1), gastric aspirate (1) | 2 (bronchial aspirate, gastric aspirate) | 0 | 20/09/2004 | *M. kansasii* | NR |
| 36 | 22/10/2008 | 4 | sputum (3), gastric aspirate (1) | 4 | 3 | 27/10/2008 | *M. kansasii* | NR |
| 37 | 17/06/2010 | 5 | bronchial aspirate (1), BAL (1), sputum (3) | 2 (bronchial aspirate, sputum) | 0 | 27/07/2010 | *M. xenopi* | NR |
| 38 | 28/09/2011 | 2 | BAL | 2 | 0 | 07/11/2011 | *M. xenopi* | epithelio-gigantocellular granuloma with caseous necrosis |
| 39 | 09/02/2012 | 4 | bronchial aspirate (1), sputum (3) | 3 (bronchial aspirate, sputum (2)) | 0 | 23/03/2012 | *M. xenopi* | not specific |
| 40 | 07/09/2007 | 7 | bronchial aspirate (1), sputum (4), BAL (2), | 7 | 3 (bronchial aspirate, BAL (2)) | 17/09/2007 | *M. xenopi* | NR |
| 41 | 28/05/2010 | 1 | lung biopsy | 1 | 1 | 07/07/2010 | *M. xenopi* | epithelio-gigantocellular granuloma with caseous necrosis |
| 42 | 01/02/2011 | 2 | bronchial aspirate (1), BAL (1) | 2 | 0 | 09/02/2011 | *M. intracellulare* | NR |
| 43 | 21/06/2011 | 1 | BAL | 1 | 1 | 27/06/2011 | *M. abscessus* | NR |
| 44 | 15/01/2009 | 2 | sputum | 2 | 0 | 22/01/2009 | *M. abscessus* | NR |
| 45 | 10/10/2007 | 2 | bronchial aspirate (1), BAL (1) | 2 | 2 | 18/10/2007 | *M. avium* | NR |
| 46 | 23/08/2003 | 5 | bronchial aspirate (1), BAL (1), sputum (3) | 5 | 4 (bronchial aspirate, sputum (3)) | 29/08/2003 | *M. avium* | NR |
| 47 | 01/12/2005 | 2 | bronchial aspirate (1), BAL (1) | 2 | 2 | 12/07/2005 | *M. avium* | not specific |
| 48 | 30/01/2004 | 2 | gastric aspirate (1), lung biopsy (1) | 2 | 0 | 17/02/2004 | *M. avium* | NR |
| 49 | 01/07/2009 | 1 | BAL | 1 | 0 | 30/07/2009 | *M. intracellulare* | NR |
| 50 | 04/11/2009 | 2 | bronchial aspirate (1), BAL (1) | 2 | 0 | 24/11/2009 | *M. intracellulare* | NR |
| 51 | 20/06/2008 | 5 | bronchial aspirate (1), BAL (1), sputum (3) | 4 (bronchial aspirate, BAL, sputum (2)) | 0 | 25/07/2008 | *M. xenopi* | NR |
| 52 | 29/01/2002 | 2 | bronchial aspirate (1), BAL (1) | 2 | 0 | 07/03/2002 | *M. xenopi* | NR |
| 53 | 18/09/2010 | 5 | bronchial aspirate (1), BAL (1), sputum (3) | 4 (BAL, sputum (3)) | 0 | 25/10/2010 | *M. xenopi* | NR |
| 54 | 04/06/2007 | 4 | sputum | 3 | 1 | 02/07/2007 | *M. xenopi* | NR |
| 55 | 21/07/2010 | 7 | bronchial aspirate (1), BAL (1), lung biopsy (1), sputum (2), gastric aspirate (2) | 5 (bronchial aspirate, BAL, gastric aspirate, sputum (2)) | 0 | 16/08/2010 | *M. xenopi* | NR |
| 56 | 26/03/2009 | 1 | BAL | 1 | 1 | 04/04/2009 | *M. intracellulare* | NR |
| 57 | 26/06/2003 | 1 | BAL | 1 | 0 | 11/07/2003 | *M. avium* | NR |
| 58 | 03/11/2003 | 5 | sputum (3), bronchial aspirate (2) | 5 | 3 | 20/11/2003 | *M. intracellulare* | NR |
| 59 | 17/03/2009 | 2 | bronchial aspirate | 2 | 0 | 30/03/2009 | *M. intracellulare* | NR |
| 60 | 26/02/2009 | 3 | BAL (1), sputum (2) | 3 | 3 | 04/03/2009 | *M. kansasii* | NR |
| 61 | 07/08/2009 | 3 | bronchial aspirate (1), BAL (2) | 3 | 1  (BAL) | 14/08/2008 | *M. simiae* | NR |
| 62 | 23/12/2010 | 1 | BAL | 1 | 0 | 29/12/2010 | *M. avium* | NR |
| 63 | 18/08/2005 | 4 | sputum (1), gastric aspirate (3) | 4 | 2 (sputum, gastric aspirate) | 25/08/2005 | *M. intracellulare* | NR |
| 64 | 17/10/2012 | 1 | BAL | 1 | 1 | 22/10/2012 | *M. intracellulare* | NR |
| 65 | 04/05/2009 | 2 | bronchial aspirate (1), BAL (1) | 2 | 0 | 11/05/2009 | *M. intracellulare* | NR |
| 66 | 28/07/2008 | 6 | bronchial aspirate (1), BAL (1), sputum (4) | 6 | 6 | 04/08/2008 | *M. kansasii* | NR |
| 67 | 06/03/2006 | 3 | BAL (1), sputum (2) | 3 | 2 (BAL, sputum) | 13/03/2006 | *M. kansasii* | NR |
| 68 | 07/02/2011 | 3 | gastric aspirate | 2 | 0 | 21/02/2011 | *M. kansasii* | NR |
| 69 | 05/10/2007 | 1 | BAL | 1 | 0 | 16/10/2007 | *M. scrofulaceum* | NR |
| 70 | 11/06/2008 | 3 | lung biopsy (1), BAL (1), sputum (1) | 1 (lung biopsy) | 0 | 15/07/2008 | *M. xenopi* | epithelio-gigantocellular granuloma with caseous necrosis |
| 71 | 01/07/2010 | 1 | lung biopsy | 1 | 1 | 26/07/2010 | *M. xenopi* | epithelio-gigantocellular granuloma with caseous necrosis |
| 72 | 29/12/2009 | 2 | bronchial aspirate (1), BAL (1) | 2 | 0 | 04/01/2010 | *M. avium* | NR |
| 73 | 15/06/2011 | 1 | bronchial aspirate | 1 | 0 | 27/06/2011 | *M. avium* | lung cancer |
| 74 | 03/01/2006 | 3 | bronchial aspirate (1), BAL (1), sputum (1) | 1 (bronchial aspirate) | 0 | 10/01/2006 | *M. avium* | NR |
| 75 | 04/11/2009 | 1 | BAL | 1 | 0 | 10/11/2009 | *M. avium* | not specific |
| 76 | 24/11/2008 | 2 | bronchial aspirate (1), BAL (1) | 2 | 0 | 04/12/2008 | *M. intracellulare* | NR |
| 77 | 31/05/2007 | 4 | bronchial aspirate (1), sputum (3) | 3 (bronchial aspirate, sputum (2)) | 0 | 11/06/2007 | *M. intracellulare* | NR |
| 78 | 15/06/2003 | 2 | bronchial aspirate (2) | 2 | 0 | 11/07/2003 | *M. intracellulare* | NR |
| 79 | 30/09/2010 | 1 | lung biopsy | 1 | 1 | 08/10/2012 | *M. intracellulare* | epithelio-gigantocellular granuloma with caseous necrosis |
| 80 | 02/10/2006 | 2 | bronchial aspirate (1), BAL (1) | 2 | 0 | 16/10/2006 | *M. kansasii* | epithelio-gigantocellular granuloma with caseous necrosis |
| 81 | 12/05/2010 | 4 | bronchial aspirate (2), BAL (2) | 1  (bronchial aspirate) | 0 | 31/05/2010 | *M. kansasii* | NR |
| 82 | 23/06/2010 | 2 | bronchial aspirate (1), BAL (1) | 2 | 0 | 05/07/2010 | *M. kansasii* | NR |
| 83 | 07/01/2009 | 2 | sputum | 2 | 0 | 16/02/2009 | *M. xenopi* | NR |
| 84 | 21/02/2005 | 1 | BAL | 1 | 0 | 10/03/2005 | *M. avium* | NR |
| 85 | 01/12/2010 | 4 | BAL (1), sputum (3) | 3 (sputum) | 0 | 08/12/2010 | *M. avium* | NR |
| 86 | 23/03/2009 | 2 | sputum (2) | 2 | 2 | 27/03/2009 | *M. intracellulare* | NR |
| 87 | 10/05/2005 | 2 | sputum (2) | 2 | 0 | 20/05/2005 | *M. intracellulare* | NR |
| 88 | 10/04/2003 | 1 | bronchial aspirate | 1 | 1 | 22/04/2003 | *M. kansasii* | NR |
| 89 | 13/09/2010 | 5 | bronchial aspirate (1), BAL (1), sputum (3) | 5 | 3 (sputum) | 29/09/2010 | *M. xenopi* | not specific |
| 90 | 01/02/2011 | 1 | BAL | 1 | 0 | 24/03/2011 | *M. xenopi* | NR |
| 91 | 27/05/2009 | 2 | bronchial aspirate (2) | 2 | 0 | 08/06/2009 | *M. avium* | NR |
| 92 | 20/01/2006 | 2 | sputum (2) | 2 | 0 | 31/01/2006 | *M. avium* | NR |

^a^ When multiple specimens were received, the number and the origin of each of them are indicated in parentheses.

^b^ The number and the origin of the positive specimens are indicated in parentheses.

BAL: bronchoalveolar lavage; NR: not realized.
