# Supplementary material for: Nontuberculous Mycobacterial Infections in a French Hospital: A 12-Year Retrospective Study
Source: PLoS One. 2016 Dec 13;11(12):e0168290. doi: 10.1371/journal.pone.0168290 (PMC5154556; doi:10.1371/journal.pone.0168290)
Supplement: S2 Table — a When multiple specimens were received, the number and the origin of each of them are indicated in parentheses. b The number and the origin of the positive specimens are indicated in parentheses. BAL: bronchoalveolar lavage; NR: not realized. (DOCX) [file pone.0168290.s002.docx]

**S2 Table. Microbiological and histological characteristics for the 78 patients with NTM extrapulmonary infection.**

| **Skin and soft-tissue infections** | | | | | | | | |
| --- | --- | --- | --- | --- | --- | --- | --- | --- |
| **Patient number** | **Sampling date** | **Number of specimens received** | **Specimens origin**^a^ | **Number of specimens with positive culture**^b^ | **Number of specimens with positive smear and positive culture**^b^ | **Date of isolation** | **Species** | **Histological results** |
| 93 | 24/02/2012 | 5 | skin abscess | 5 | 0 | 12/03/2012 | *M. abscessus* | NR |
| 94 | 22/05/2008 | 2 | skin biopsy | 1 | 0 | 30/05/2008 | *M. avium* | epithelio-gigantocellular granuloma with caseous necrosis |
| 95 | 14/01/2011 | 1 | skin biopsy | 1 | 1 | 18/01/2011 | *M. chelonae* | epithelio-gigantocellular granuloma with caseous necrosis |
| 96 | 22/04/2009 | 1 | skin biopsy | 1 | 0 | 04/05/2009 | *M. chelonae* | not specific |
| 97 | 08/07/2008 | 1 | skin biopsy | 1 | 1 | 15/07/2008 | *M. chelonae* | epithelio-gigantocellular granuloma with caseous necrosis |
| 98 | 22/01/2009 | 1 | skin biopsy | 1 | 0 | 09/02/2009 | *M. chelonae* | not specific |
| 99 | 02/05/2011 | 1 | synovial biopsy | 1 | 0 | 30/04/2011 | *M. intracellulare* | epithelio-gigantocellular granuloma with caseous necrosis |
| 100 | 20/12/2012 | 1 | skin biopsy | 1 | 0 | 14/01/2013 | *M. intracellulare* | epithelio-gigantocellular granuloma with caseous necrosis |
| 101 | 05/01/2011 | 1 | skin biopsy | 1 | 0 | 31/01/2011 | *M. marinum* | epithelio-gigantocellular granuloma with caseous necrosis |
| 102 | 10/02/2010 | 1 | synovial biopsy | 1 | 0 | 25/02/2010 | *M. marinum* | epithelio-gigantocellular granuloma without caseous necrosis |
| 103 | 11/10/2006 | 1 | skin biopsy | 1 | 0 | 13/11/2006 | *M. marinum* | epithelio-gigantocellular granuloma with caseous necrosis |
| 104 | 18/02/2011 | 1 | skin biopsy | 1 | 0 | 15/03/2011 | *M. marinum* | not specific |
| 105 | 30/10/2012 | 1 | skin biopsy | 1 | 0 | 03/12/2012 | *M. marinum* | not specific |
| 106 | 24/11/2007 | 1 | skin biopsy | 1 | 0 | 18/12/2007 | *M. marinum* | not specific |
| 107 | 26/04/2007 | 1 | skin biopsy | 1 | 0 | 09/05/2007 | *M. marinum* | not specific |
| 108 | 14/03/2008 | 2 | synovial biopsy | 1 | 0 | 31/03/2008 | *M. marinum* | epithelio-gigantocellular granuloma without caseous necrosis |
| 109 | 04/01/2012 | 1 | skin biopsy | 1 | 0 | 16/01/2012 | *M. marinum* | NR |
| 110 | 27/05/2011 | 1 | skin biopsy | 1 | 0 | 20/06/2011 | *M. marinum* | epithelio-gigantocellular granuloma without caseous necrosis |
| 111 | 06/04/2012 | 1 | skin biopsy | 1 | 0 | 27/05/2012 | *M. szulgaï* | epithelio-gigantocellular granuloma with caseous necrosis |
| 112 | 31/10/2012 | 1 | skin biopsy | 1 | 0 | 23/11/2012 | *M. avium* | epithelio-gigantocellular granuloma with caseous necrosis |
| 113 | 20/09/2006 | 1 | fistula | 1 | 1 | 25/09/2006 | *M. intracellulare* | NR |
| 114 | 03/12/2008 | 1 | synovial biopsy | 1 | 0 | 18/12/2008 | *M. intracellulare* | epithelio-gigantocellular granuloma without caseous necrosis |
| 115 | 12/07/2006 | 1 | skin biopsy | 1 | 0 | 24/07/2006 | *M. marinum* | not specific |
| 116 | 02/12/2004 | 1 | synovial biopsy | 1 | 0 | 13/12/2004 | *M. terrae* | epithelio-gigantocellular granuloma without caseous necrosis |
| 117 | 18/11/2005 | 1 | synovial biopsy | 1 | 0 | 05/12/2005 | *M. marinum* | epithelio-gigantocellular granuloma without caseous necrosis |
| 118 | 18/07/2012 | 1 | skin biopsy | 1 | 0 | 06/08/2012 | *M. marinum* | NR |
| 119 | 07/05/2007 | 1 | skin biopsy | 1 | 0 | 25/05/2007 | *M. marinum* | not specific |
| 120 | 25/02/2003 | 1 | skin biopsy | 1 | 0 | 10/03/2003 | *M. marinum* | epithelio-gigantocellular granuloma without caseous necrosis |
| 121 | 17/02/2006 | 1 | skin biopsy | 1 | 0 | 06/03/2006 | *M. marinum* | epithelio-gigantocellular granuloma without caseous necrosis |
| 122 | 20/10/2006 | 2 | synovial biopsy | 2 | 0 | 26/12/2006 | *M. intracellulare* | not specific |
| 123 | 09/05/2008 | 1 | skin biopsy | 1 | 0 | 06/06/2008 | *M. marinum* | NR |
| 124 | 23/03/2009 | 1 | synovial biopsy | 1 | 0 | 17/04/2009 | *M. marinum* | NR |
| 125 | 06/02/2007 | 1 | skin biopsy | 1 | 0 | 26/02/2007 | *M. marinum* | epithelio-gigantocellular granuloma without caseous necrosis |
| 126 | 03/06/2010 | 1 | skin biopsy | 1 | 0 | 28/06/2010 | *M. marinum* | NR |
| 127 | 13/07/2007 | 1 | skin biopsy | 1 | 0 | 31/07/2007 | *M. marinum* | epithelio-gigantocellular granuloma without caseous necrosis |
| 128 | 28/09/2010 | 1 | skin biopsy | 1 | 0 | 02/11/2010 | *M. marinum* | epithelio-gigantocellular granuloma without caseous necrosis |
| 129 | 25/09/2009 | 1 | synovial biopsy | 1 | 0 | 28/10/2009 | *M. xenopi* | NR |
| 130 | 11/03/2005 | 1 | skin biopsy | 1 | 0 | 09/05/2005 | *M. marinum* | epithelio-gigantocellular granuloma without caseous necrosis |
| 131 | 28/01/2009 | 1 | skin biopsy | 1 | 0 | 02/02/2009 | *M. chelonae* | epithelio-gigantocellular granuloma without caseous necrosis |

| **Lymphadenitis** | | | | | | | | |
| --- | --- | --- | --- | --- | --- | --- | --- | --- |
| **Patient number** | **Sampling date** | **Number of specimens received** | **Specimens origin**^a^ | **Number of specimens with positive culture**^b^ | **Number of specimens with positive smear and positive culture**^b^ | **Date of isolation** | **Species** | **Histological results** |
| 132 | 22/06/2009 | 1 | submandibular lymph node biopsy | 1 | 1 | 29/06/2009 | *M. avium* | epithelio-gigantocellular granuloma with caseous necrosis |
| 133 | 28/09/2012 | 1 | submandibular lymph node biopsy | 1 | 0 | 08/10/2012 | *M. avium* | NR |
| 134 | 18/05/2007 | 1 | submandibular lymph node biopsy | 1 | 0 | 30/05/2007 | *M. avium* | epithelio-gigantocellular granuloma without caseous necrosis |
| 135 | 16/07/2008 | 1 | cervical lymph node biopsy | 1 | 0 | 22/07/2008 | *M. avium* | not specific |
| 136 | 17/02/2012 | 1 | submandibular lymph node biopsy | 1 | 0 | 01/03/2011 | *M. avium* | NR |
| 137 | 12/05/2010 | 1 | submandibular lymph node biopsy | 1 | 0 | 18/05/2012 | *M. avium* | epithelio-gigantocellular granuloma without caseous necrosis |
| 138 | 04/01/2012 | 1 | cervical lymph node biopsy | 1 | 0 | 16/01/2012 | *M. avium* | not specific |
| 139 | 30/06/2009 | 1 | submandibular lymph node biopsy | 1 | 0 | 06/07/2009 | *M. avium* | epithelio-gigantocellular granuloma with caseous necrosis |
| 140 | 27/10/2010 | 2 | submandibular and preauricular lymph nodes biopsies | 2 | 2 | 02/11/2010 | *M. avium* | not specific |
| 141 | 11/04/2007 | 2 | cervical lymph node biopsy | 2 | 0 | 23/04/2007 | *M. avium* | NR |
| 142 | 18/04/2008 | 1 | parotid lymph node biopsy | 1 | 0 | 13/05/2008 | *M. avium* | NR |
| 143 | 04/02/2012 | 2 | axillary lymph node biopsy | 1 | 0 | 16/02/2012 | *M. avium* | not specific |
| 144 | 29/07/2004 | 1 | lymph node biopsy | 1 | 1 | 16/08/2004 | *M. kansasii* | epithelio-gigantocellular granuloma without caseous necrosis |

| **Bone and joint infections** | | | | | | | | |
| --- | --- | --- | --- | --- | --- | --- | --- | --- |
| **Patient number** | **Sampling date** | **Number of specimens received** | **Specimens origin**^a^ | **Number of specimens with positive culture**^b^ | **Number of specimens with positive smear and positive culture**^b^ | **Date of isolation** | **Species** | **Histological results** |
| 145 | 17/01/2012 | 2 | joint fluid | 1 | 0 | 30/01/2012 | *M. abscessus* | epithelio-gigantocellular granuloma without caseous necrosis |
| 146 | 09/05/2011 | 4 | knee bone biopsy | 4 | 0 | 12/05/2011 | *M. chelonae* | NR |
| 147 | 17/03/2006 | 1 | calcaneus biopsy | 1 | 0 | 27/03/2006 | *M. chelonae* | epithelio-gigantocellular granuloma without caseous necrosis |
| 148 | 30/07/2010 | 1 | prosthetic joint biopsy | 1 | 0 | 06/09/2010 | *M. intracellulare* | NR |
| 149 | 12/11/2002 | 2 | joint fluid (1), sputum (1) | 2 | 0 | 02/12/2002 | *M. kansasii* | NR |

| **Disseminated infections** | | | | | | | | |
| --- | --- | --- | --- | --- | --- | --- | --- | --- |
| **Patient number** | **Sampling date** | **Number of specimens received** | **Specimens origin**^a^ | **Number of specimens with positive culture**^b^ | **Number of specimens with positive smear and positive culture**^b^ | **Date of isolation** | **Species** | **Histological results** |
| 150 | 28/01/2011 | 4 | blood (4) | 4 | NR | 07/02/2011 | *M. avium* | NR |
| 151 | 25/01/2012 | 9 | blood (3), gastric aspirate (3), BAL (1), bronchial aspirate (1), rectal biopsy (1) | 8 (3 blood, 3 gastric aspirate, 1 BAL, 1 rectal biopsy) | 0 | 02/02/2012 | *M. avium* | NR |
| 152 | 24/04/2010 | 7 | sputum (3), blood (4) | 2 (blood, sputum) | 0 | 17/05/2010 | *M. avium* | NR |
| 153 | 07/08/2008 | 6 | sputum (4), blood (2) | 1  (blood) | 0 | 08/09/2008 | *M. intracellulare* | NR |
| 154 | 05/08/2005 | 7 | sputum (3), blood (4) | 6 (sputum (2), blood (4)) | 0 | 17/08/2005 | *M. simiae* | NR |
| 155 | 09/09/2004 | 2 | blood | 2 | NR | 21/09/2004 | *M. avium* | NR |
| 156 | 22/06/2004 | 8 | blood | 8 | NR | 15/07/2004 | *M. avium* | NR |
| 157 | 01/03/2006 | 5 | blood (4), bone marrow (1) | 5 | NR | 10/03/2006 | *M. avium* | epithelio-gigantocellular granuloma without caseous necrosis |
| 158 | 14/02/2007 | 6 | bone marrow (1), blood (5) | 6 | NR | 26/02/2007 | *M. avium* | NR |
| 159 | 13/06//2009 | 7 | blood (3), liver biopsy (1), bone marrow (1), sputum (2) | 2 (blood) | 0 | 20/07/2009 | *M. genavense* | epithelio-gigantocellular granuloma without caseous necrosis |
| 160 | 11/08/2010 | 4 | blood (1), bone marrow (1), BAL (1), bronchial aspirate (1) | 2 (blood, BAL) | 0 | 23/08/2012 | *M. avium* | NR |
| 161 | 01/07/2003 | 3 | bone marrow, blood (2) | 2 (blood) | NR | 15/07/2003 | *M. avium* | NR |
| 162 | 27/05/2003 | 4 | blood (4) | 4 | NR | 10/06/2003 | *M. avium* | NR |
| 163 | 09/02/2012 | 5 | blood (2), bronchial aspirate (2), lodge of the pace maker (1) | 4 (blood, bronchial aspirate (2), lodge of pace-maker) | 2 (bronchial aspirate) | 14/02/2012 | *M. chelonae* | NR |
| 164 | 26/02/2011 | 7 | blood (1), gastric aspirate (2), sputum (2), bronchial aspirate (2) | 1  (blood) | 0 | 20/04/2011 | *M. simiae* | NR |
| 165 | 22/02/2010 | 1 | cerebrospinal fluid | 1 | NR | 04/03/2010 | *M. avium* | NR |
| 166 | 29/01/2003 | 2 | blood (1), BAL (1) | 2 | 0 | 14/02/2003 | *M. avium* | NR |
| 167 | 06/03/2005 | 1 | blood | 1 | NR | 16/03/2005 | *M. avium* | NR |

| **Catheter-related infection** | | | | | | | | |
| --- | --- | --- | --- | --- | --- | --- | --- | --- |
| **Patient number** | **Sampling date** | **Number of specimens received** | **Specimens origin**^a^ | **Number of specimens with positive culture**^b^ | **Number of specimens with positive smear and positive culture**^b^ | **Date of isolation** | **Species** | **Histological results** |
| 168 | 01/02/2011 | 1 | port | 1 | 1 | 02/02/2011 | *M. chelonae* | NR |
| 169 | 09/01/2010 | 2 | intracardiac leads (2) | 2 | 0 | 18/10/2010 | *M. fortuitum* | NR |
| 170 | 10/07/2009 | 2 | port, port-a-cath | 2 | 2 | 15/07/2009 | *M. chelonae* | NR |

^a^ When multiple specimens were received, the number and the origin of each of them are indicated in parentheses.

^b^ The number and the origin of the positive specimens are indicated in parentheses.

BAL: bronchoalveolar lavage; NR: not realized.
